# Interaction Between Telocytes and Mast Cells in Genetically Determined Non-Obstructive Azoospermia with AZFc Deletion: An Ultrastructural Study

**DOI:** 10.3390/ijms27072923

**Published:** 2026-03-24

**Authors:** Irina Chekmareva, Andrey Kostin, Nina Kulchenko, Grigory Demyashkin, Oksana Paklina, Alexander Alekhnovich, Artem Volodkin, Atim Emaimo John, Ilya Klabukov, Denis Baranovskii, Viktoria Shishkina, Igor Buchwalow, Markus Tiemann, Dmitrii Atiakshin

**Affiliations:** 1 Federal State Budgetary Institution “National Medical Research Center of Surgery Named After A.V. Vishnevsky” of the Ministry of Health of the Russian Federation, Bolshaya Serpukhovskaya St., 27, Moscow 115093, Russia; chia236@mail.ru (I.C.); dr.oxanapaklina@mail.ru (O.P.); 2 Research and Educational Resource Center for Immunophenotyping, Digital Spatial Profiling and Ultrastructural Analysis Innovative Technologies, RUDN University, 6 Miklukho-Maklaya St., Moscow 117198, Russia; andocrey@mail.ru (A.K.); kle-kni@mail.ru (N.K.); dr.dga@mail.ru (G.D.); alekhnovich-av@rudn.ru (A.A.); volodkin-av@rudn.ru (A.V.); emaimo-dzhon-a@rudn.ru (A.E.J.); buchwalow@pathologie-hh.de (I.B.); 3 State Budgetary Healthcare Institution “Moscow Clinical Scientific Center Named After A.S. Loginov, Department of Health of the City of Moscow”, Enthusiasts Highway, 86, Moscow 111123, Russia; 4 Department of Regenerative Medicine, National Medical Research Radiological Centre of the Ministry of Health of the Russian Federation, Koroleva St., Obninsk 249036, Russiadoc.baranovsky@gmail.com (D.B.); 5 Research Institute of Experimental Biology and Medicine, Burdenko Voronezh State Medical University, Voronezh 394036, Russia; 4128069@gmail.com; 6 Institute for Hematopathology, Fangdieckstr. 75a, 22547 Hamburg, Germany; mtiemann@hp-hamburg.de

**Keywords:** male infertility, AZFc deletion, mast cells, telocytes, electron microscopy, fibrosis, collagen, extracellular matrix, fibers, ultrastructural features

## Abstract

In idiopathic azoospermia caused by non-obstructive infertility with AZFc deletion, the testicle usually contains an increased number of mast cells (MCs)—which are responsible for collagen synthesis in the testes—as well as Leydig cell hyperplasia. However, the relationship between MCs and telocytes in this pathology remains unexplored. The aim of this study was to examine ultrastructural changes in the interstitial tissue microenvironment of the convoluted seminiferous tubules in the testis, using clinical specimens from men with genetically determined non-obstructive infertility with AZFc deletion. Histological, immunohistochemical, and electron microscopic (EM) studies were performed on surgical materials from 14 patients with AZFc deletion. The IHC study was performed using a panel of antibodies: tryptase, chymase, carboxypeptidase A3, and αSMA. The EM study was performed on ultrathin sections with a thickness of 100–120 nm. MCs were found to be in a functionally active state and characterized by a variety of secretory activities. For the first time, telocytes and their colocalization with MCs and Leydig cells were visualized. It is possibly the telocytes—interacting with MCs—that synchronize the functional activity of the entire MC population of the testis. The interaction of MCs with telocytes, as well as individual secretory granules associated with loci of tropocollagen and collagen microfibril accumulation, leads to the accumulation of collagen fibrils in the interstitium, as observed in idiopathic infertility with AZFc deletion. Even with a small number of MCs in the interstitium of the convoluted seminiferous tubules in the testis, the telocytes are able to synchronize MCs’ activation and secretory activity, supporting the development of a profibrotic phenotype of the tissue microenvironment. The obtained results advance our understanding of idiopathic infertility with AZFc deletion by delineating the ultrastructural landscape of the testicular interstitium and establishing telocytes as key regulators of cellular crosstalk. Telocytes use complex mechanisms for the spatial integration of MCs and fibroblasts in the profibrotic phenotype formation of the convoluted seminiferous tubule tissue microenvironment. Potentially, telocytes can directly be involved in synchronizing such processes by activating the biogenesis and secretion of collagen monomers by fibroblasts; the MC secretome directly affects the polymerization of collagen monomers and dimers into microfibrils in the extracellular matrix, stimulating excessive collagen fiber formation and the development of fibrotic changes.

## 1. Introduction

Male infertility is recognized as a polyetiological disease [1,2,3,4,5]. Male fertility disorders can be caused by hypothalamic–pituitary system disorders, cryptorchidism, varicocele, malignant neoplasms of the testicles, trauma, genetic abnormalities, inflammatory diseases of the testicles of various origins, etc. [6,7,8,9,10,11]. Male infertility is classified as idiopathic in about one-third of cases, most of which may be due to genetic factors, such as various chromosomal abnormalities or mutations in the genes responsible for spermatogenesis [12,13,14].

The role of Yq11 locus deletions in the etiology of spermatogenesis disorders and male infertility was first demonstrated in 1976 by L. Tiepolo and O. Zuffardi [15]. Further cytogenetic and molecular genetic studies using STS (sequence-tagged site) technology led to the construction of a detailed Y chromosome map, including 43 deletion intervals [16]. The AZF locus was confirmed to be in the distal part of the Y chromosome long arm. AZFc deletion results in impaired spermatogenesis. In 1995, using data on the location and size of deletions in a group of 26 male patients, P.H. Vogt et al. distinguished three non-overlapping subregions in the Yq11.21-q11.23 locus: AZFa, AZFb, and AZFc [17]. Individuals with deletions affecting the AZFa or AZFb subregions are unable to produce mature germ cells, while in patients with AZFc deletions, mature spermatozoa can be obtained during testicular biopsy in approximately 50–70% of cases [18,19].

In specific tissue microenvironments, the role of mast cells (MCs) in the regulation of local homeostasis is crucial [20,21,22]. On the one hand, MCs express a wide range of receptors that provide highly sensitive mechanisms for the formation of a selective response to external and internal impact. On the other hand, MCs can selectively secrete various classes of mediators and alternative cytokine and chemokine profiles, exerting various biological effects [23,24]. MC tools are three main classes of mediators: preformed mediators, mediators of lipid origin, and multiple cytokines, chemokines, and growth factors formed after MC stimulation for the necessary modification of physiological reactions and immune functions [25,26]. MCs play a special role in the development of the pro-inflammatory background, regulating the state of numerous cells of the immune and stromal landscape, as well as the extracellular matrix of connective tissues [27,28,29,30,31,32,33,34,35].

A number of studies have noted the role of mast cells (MCs) in the development of idiopathic non-obstructive male infertility [36,37]. Normally, MCs play a regulatory role in maintaining testicular homeostasis and are an integral component of testicular tissue. According to a number of authors, pro-inflammatory cytokines secreted by MCs—interleukins (ILs) 1 and 6 and tumor necrosis factor alpha (TNFα)—affect the differentiation of spermatogenic epithelial cells, steroidogenesis, and germ cell apoptosis in the testis under physiological conditions [21,22,38,39,40,41]. According to Haidl et al. (2011), an increase in the MC population in the testicle interstitial tissue and the contact of cells with the convoluted seminiferous tubules may cause dysfunction of the blood–testis barrier, which can impair fertility [41,42,43]. MCs contribute to the development of chronic inflammatory reactions in the testis and also affect the reparative regeneration of the testicles [39,44,45,46,47]. Several reports have noted a correlation between elevated MC levels in the testes and male infertility [48,49,50,51,52]. For example, Nagai et al. (1992) found that MC levels increase in idiopathic azoospermia and oligozoospermia [48,49,50]. Since 1995, MC blockers have been used to treat male infertility, with a significant improvement in sperm quality [53,54,55]. However, some papers provide conflicting results [56].

Telocytes (TCs) play a key role in regulating the local tissue microenvironment [57]. Possessing a unique morphological structure, TCs form a three-dimensional network, facilitating the organization of the non-cellular matrix, paracrine and extracrine communication, immunogenesis, immune surveillance, cell survival, apoptosis, etc. [58]. TCs have been detected in the interstitial tissue of the testes in many animal species [59,60,61].

The pathogenesis of genetically determined male infertility with AZFc deletion at the level of the local tissue niche microenvironment remains unclear. However, there is no doubt that the interaction between mast cells and TCs is the most important factor among the mechanisms for maintaining homeostasis of the immune and stromal landscape of the gonads. To obtain new data on the mechanisms regulating local homeostasis in the stroma of the convoluted seminiferous tubules, including mast cell and TC involvement, we analyzed ultrastructural changes in the convoluted seminiferous tubules in the testes of men with genetically determined non-obstructive infertility with AZFc deletion.

## 2. Results

All the patients studied (*n* = 14) had a male phenotype: a correct body structure, satisfactory development of secondary sexual characteristics (male-type hair growth), and intact sexual function. The average age of all patients was 31.2 ± 8.3 years. The body mass index of all patients was 29.1 ± 4.8, indicating overweight. The testicles were soft upon palpation of the scrotum. The average testicular volume in patients with AZFc deletion (according to ultrasound data) was 10.4 ± 1.9 mL, which was significantly lower than the reference values (*p* = 0.04). According to the spermogram, all patients had non-obstructive azoospermia, which means that there were no sperm cells in the semen after centrifugation. The hormonal profiles of the patients were characterized by a significant increase in the level of follicle-stimulating hormone (FSH) to 17.2 ± 1.9 mIU/mL, with reference values of 1.5–12.4 mIU/mL (*p* < 0.01). The hypergonadotropic status was accompanied by a significant decrease in the inhibin B level, which averaged 76.9 ± 21.7 pg/mL (*p* < 0.01 compared to the lower limit of the normal range of 147.0 pg/mL), reflecting severe damage to the spermatogenic epithelium and impaired function of the Sertoli cells. The levels of luteinizing hormone (LH), total testosterone, sex hormone-binding globulin (SHBG), prolactin, and estradiol were within the reference intervals and did not have statistically significant deviations (*p* > 0.05). Detailed characteristics of the hormonal indicators are presented in Table 1.

Thus, the examined cohort of patients demonstrates a clinico-hormonal phenotype characteristic of AZFc deletion: non-obstructive azoospermia combined with a moderate decrease in testicular volume, a compensatory increase in FSH, and a significant decrease in the level of inhibin B, which indicates a profound impairment of testicular secretory function.

**Figure 1:**
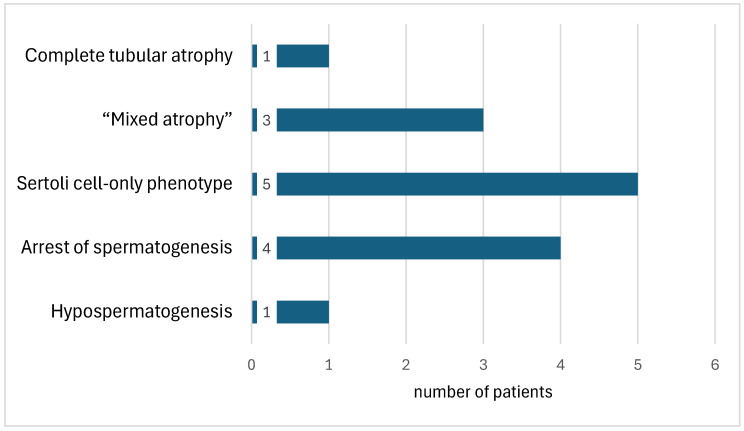
Histopathological disorders of spermatogenesis in patients with AZFc deletion of the Y chromosome.

A qualitative morphological analysis of hematoxylin-and-eosin-stained testicular sections showed that patients with AZFc-Y chromosome deletion most often had the Sertoli cell-only phenotype (35.7%). Arrest of spermatogenesis (28.6%) and mixed atrophy (21.4%) were also observed (Figure 1).

**Figure 2:**
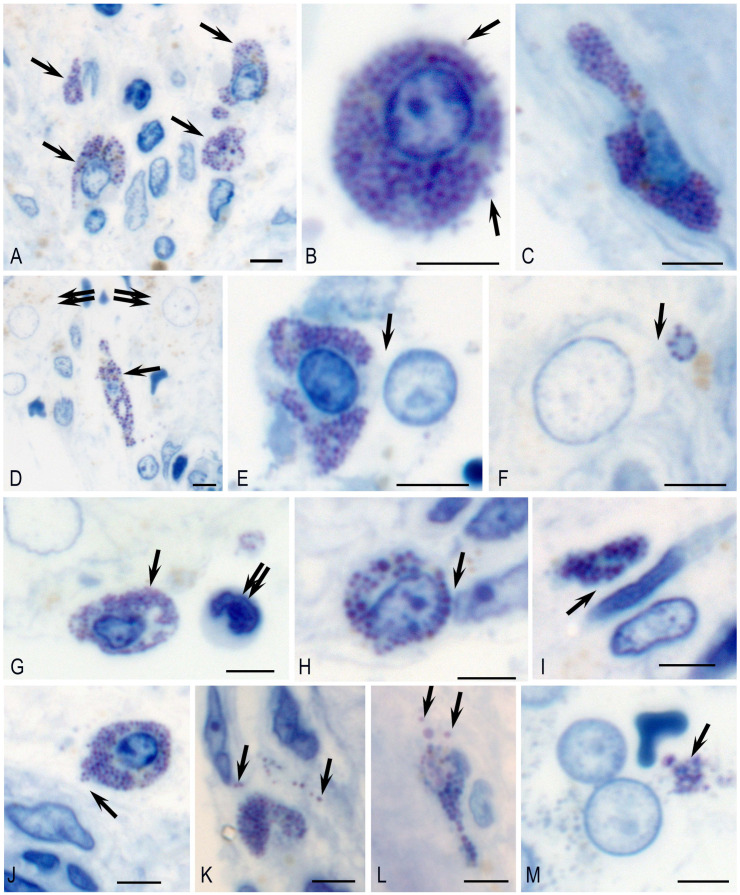
Identification and localization of mast cells in the testicular interstitium. Methods: Histological staining of semi-thin sections with toluidine blue: (**A**) Focal accumulation of morphologically heterogeneous MCs (arrow) within the testicular interstitium. (**B**) A rounded MC (arrow) exhibiting granule exocytosis in the stromal compartment. (**C**) An elongated MC within the tunica propria of a seminiferous tubule. (**D**) Paracrine colocalization of an MC (arrow) with a cluster of Leydig cells (double arrow). (**E**,**F**) Juxtacrine interactions between large (**E**) and small (**F**) MCs (arrow) and adjacent Leydig cells. (**G**) An MC (arrow) unevenly packed with secretory granules, located near a monocyte (presumed, double arrow) along the tubule wall. (**H**) Juxtacrine association between an MC and a stromal cell (arrow, presumed fibrocyte). (**I**) Colocalization of an MC and a myoid cell in the peritubular layer (arrow). (**J**–**M**) Focal secretion of MC granules (arrows): directed toward the wall of the seminiferous tubule (**J**–**L**) and toward a Leydig cell (**M**). Scale bar: 5 µm.

Toluidine-blue-stained semi-thin sections revealed that MCs were unevenly distributed in the testicular interstitium, with focal stromal accumulations (Figure 2A). The MC morphology varied with location: round shapes predominated in the intertubular stroma (Figure 2B,G,H,J,K), while an elongated phenotype was characteristic of MCs within the tubular walls (Figure 2C,D,L).

MCs exhibited targeted intercellular cooperation within the testicular interstitium. Specifically, we observed both juxtacrine and paracrine interactions with Leydig cells (Figure 2D–F). Notably, some of these interactions involved very small MCs containing perinuclear metachromatic granules in close association with Leydig cells (Figure 2F). MCs were found in various configurations with immunocompetent cells, including monocytes (Figure 2G), and, more frequently, with stromal cells, such as fibrocytes, fibroblasts, and myoid cells (Figure 2H–J). Furthermore, targeted secretory activity toward specific cellular or extracellular structures was evident in virtually every mast cell (Figure 2J–M).

**Figure 3:**
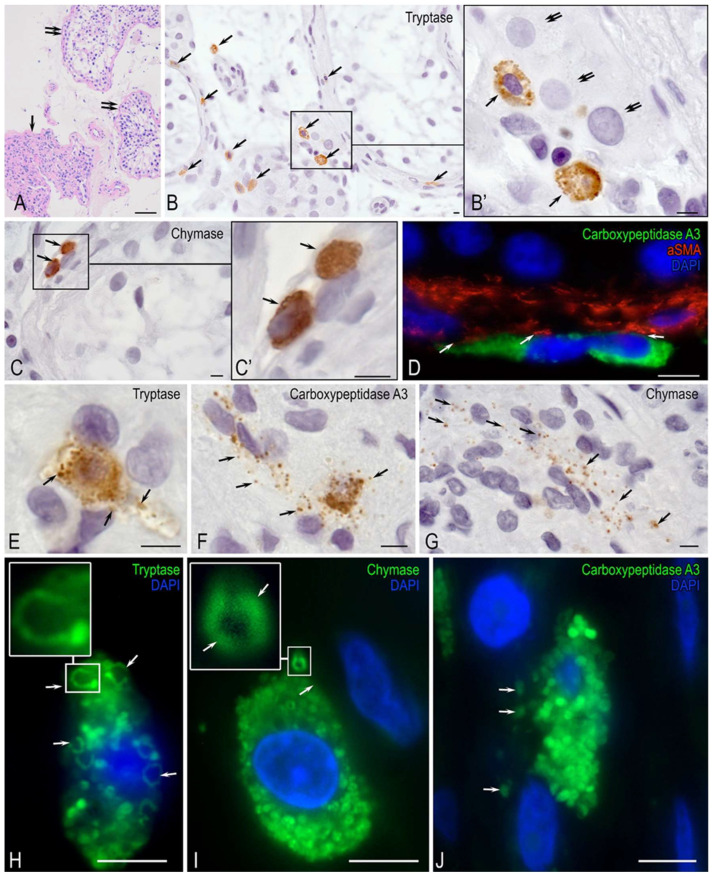
Localization and secretion of MC proteases in testicular tissue with AZFc deletion. Methods: (**A**) hematoxylin and eosin staining; (**B**,**C**,**E**–**J**) immunohistochemical detection of MC proteases visualized with DAB chromogen (**B**,**C**,**E**–**G**) or Alexa Fluor 488 (**H**–**J**); (**D**) dual immunofluorescence staining for carboxypeptidase A3 (CPA3, Alexa Fluor 488, green) and α-smooth muscle actin (α-SMA, Alexa Fluor 555, red). Comments: (**A**) Seminiferous tubule showing focal fibrosis (arrow). Multiple tubules contain spermatids with round nuclei and condensed chromatin (double arrow). The interstitium lacks marked fibrosis, and Leydig cells are not visualized. (**B**) Focal accumulation of tryptase-positive MCs in the interstitium (arrow); (**B′**) a higher-magnification view of (**B**), illustrating MC (arrow) adjacent to Leydig cells (double arrow). (**C**) Chymase-positive MCs within the tubule wall (arrow); (**C′**) enlarged view of (**C**). (**D**) Close association between CPA3^+^ MCs and α-SMA^+^ myoid cells in the peritubular region (arrow). (**E**–**G**) Extracellular release of tryptase^+^ (**E**), CPA3^+^ (**F**), and chymase^+^ (**G**) granules into the interstitial stroma (arrows). (**H**) Chymase secretion from an MC showing progressive granule depletion (arrow). (**I**) A large chymase^+^ MC exhibiting pronounced peripheral localization of protease within granules (arrow). (**J**) Wholesale release of CPA3^+^ granules into the extracellular matrix (arrow). Scale bar: 5 µm.

Light microscopy of histochemically stained sections revealed focal fibrosis in occasional seminiferous tubules (Figure 3A). Most tubules exhibited thin walls and spermatid retention, indicating impaired spermatogenesis. This was characterized by numerous spermatids with round nuclei and highly condensed chromatin (Figure 3A).

Immunohistochemical detection of specific proteases showed focal clusters of tryptase^+^, chymase^+^, and CPA3^+^ MCs (Figure 3B), some of which were found interacting with Leydig cells (Figure 3B’). Elongated protease-positive MCs were typically located within the seminiferous tubule walls, where they were associated with myoid cells (Figure 3C,D).

During active secretion, MC granules were dispersed across extensive areas of the seminiferous tubule stroma (Figure 3E–G). This exocytotic activity resulted in significant granule depletion within the cells (Figure 3H).

Immunohistochemical analysis revealed that specific proteases were localized predominantly at the periphery of large granules, with minimal staining in the central region (Figure 3I,J).

**Figure 4:**
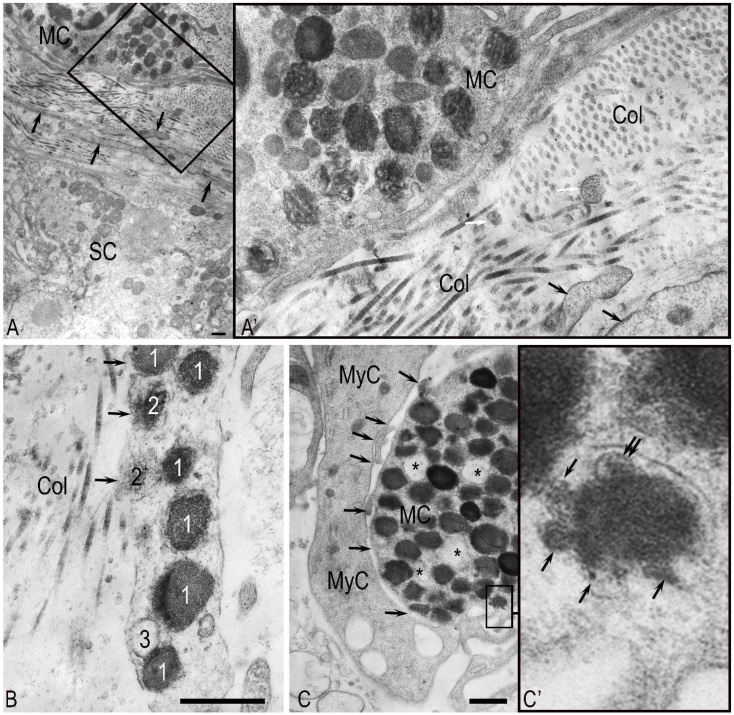
Ultrastructural analysis of MCs in the peritubular compartment: (**A**) Electron micrograph of an MC adjacent to a Sertoli cell (SC). Note the surrounding collagen fibers (Col) and processes of myoid cells (black arrows). (**A’**) A higher-magnification view of the area outlined in panel (**A**) shows MC secretory granules of diverse ultrastructures, with several (white arrows) present in the intercellular space among collagen fibrils (Col). Black arrows indicate myoid cell (MyC) processes. (**B**) An MC undergoing piecemeal degranulation, showing (**1**) electron-dense granules; (**2**) granules with partially released contents; (**3**) empty granule membranes. Note the absence of the plasma membrane at the site of granule release (black arrow). Col, collagen. (**C**) Interaction between an MC and a myoid cell (MyC). Arrows indicate contact points. The MC contains a mixture of electron-dense and electron-lucent secretory granules, the latter of which appear nearly empty (asterisk). (**C’**) A higher-magnification view of the area in (**C**), showing active degranulation with secretome release (arrow) and the formation of exosomes (presumed, double arrow). Method: Transmission electron microscopy. Scale bar: 1 µm.

During electron microscopic examination, elongated MCs were frequently detected within the seminiferous tubule walls, nestled between the processes of myoid cells and collagen fibers. MC cytoplasm contained polymorphic granules, with numerous thin cytoplasmic projections from the cell surfaces (Figure 4A). The partial or complete degranulation found in a substantial proportion of these cells is consistent with a functionally active phenotype (Figure 4B).

Rounded MCs, densely packed with secretory granules, were also present. The granules’ ultrastructure was highly heterogeneous, ranging from dense, homogeneous types with coarse- or fine-grained contents to granules with a moderate-density matrix containing denser inclusions, reticular or scroll-like formations, compact electron-dense forms, and light, vacuolated varieties. Multiple structural variants could coexist within a single granule (Figure 4A’).

MCs displayed multiple secretion mechanisms, frequently exhibiting a piecemeal (or “pacemaker”) degranulation pattern. This sustained a baseline level of proteases and other bioactive molecules in the extracellular matrix, thereby modulating the cellular and non-cellular components of the testicular microenvironment (Figure 4B).

Furthermore, MCs exhibited granule release via focal plasma membrane dissolution. We observed that MCs were surrounded by collagen microfibrils, with their released granule contents making direct contact with the matrix. Notably, MCs formed direct junctions with peritubular myoid cells, the key effectors of tubular peristalsis, suggesting a potential role in modulating contractility (Figure 4C,C’).

**Figure 5:**
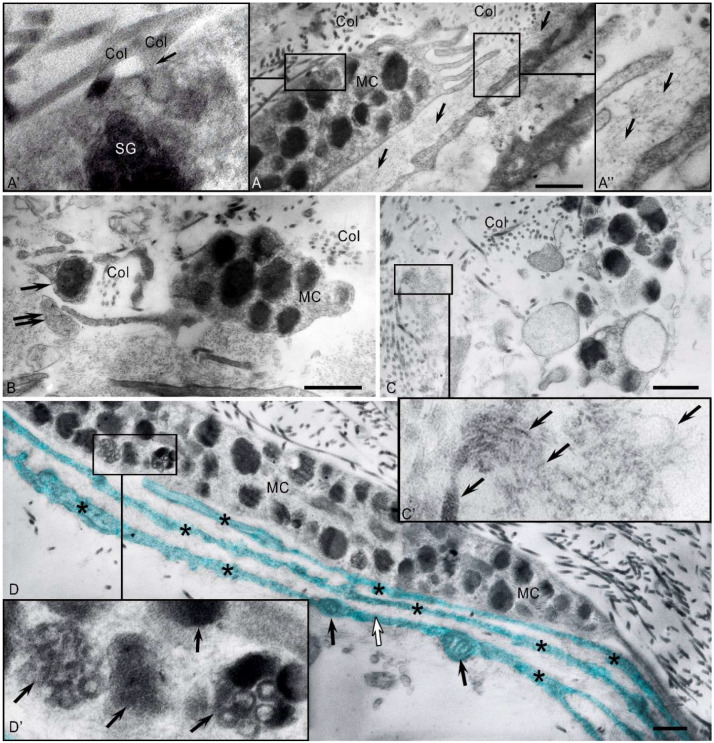
Ultrastructural features of interstitial tissue of convoluted seminiferous tubules in AZFc deletion: (**A**) An MC containing secretory granules and extending long cytoplasmic processes toward collagen fibrils (Col). Black arrows indicate granular amorphous material with thin microfibrils. (**A’**) A higher-magnification view of the area outlined in panel (**A**), showing MC secretory granules (SG). The interface between collagen microfibrils and an MC reveals active granule secretion into the intercellular space (arrow). (**A”**) A detailed view of the region indicated in (**A**), where thin collagen microfibrils are evident (arrow). (**B**) An MC surrounded by collagen fibrils (Col). Arrows indicate granules in the intercellular space: a granule within a cytoplasmic fragment (arrow) and an emptied granule with an intact membrane (double arrow). (**C**) Structural diversity of MC granules adjacent to collagen fibrils (Col). (**C’**) An enlarged fragment (**C**) in which collagen fibrillogenesis occurs (arrow). (**D**) An MC with polymorphic granules contacting TC telopodes (blue overlay, asterisk). The black arrow shows a podomer (dilated telopode segment containing mitochondria); the white arrow indicates a podom (thin segment). (**D’**) A higher-magnification view of the area outlined in panel (**D**), revealing secretory granules of heterogeneous morphology. Methods: All panels are transmission electron micrographs. Scale bar: 1 µm.

Within the interstitial tissue, MCs were surrounded by a heterogeneous extracellular matrix, comprising collagen fibrils (both striated and non-striated), thin microfibrils, and granular amorphous material. Figure 5A illustrates a mast cell interfacing with distinct matrix components: individual collagen fibrils on one side and granular amorphous material with thin microfibrils on the other, suggesting a pattern of guided migration through this stromal environment.

In addition to the secretion mechanisms previously described, we observed a “kiss-and-run” process, characterized by the transient fusion of a secretory granule with the plasma membrane. This event forms a temporary pore, enabling the direct release of proteases and other mediators into the extracellular space (Figure 5A’).

Moreover, we observed the release of individual mast cell granules or their aggregates within autonomous cytoplasmic fragments (Figure 5B). Following the partial or complete release of these fragments into the extracellular space, we detected both free granules and their limiting membranes, suggesting a mechanism of intracellular granule lysis (Figure 5B). This morphology is consistent with a “shuttle” secretion mechanism, whereby microvesicles containing proteases, proteoglycans, and other mediators bud from the parent granule to exert selective, localized effects on the tissue microenvironment.

Large autonomous MC fragments in the extracellular space exhibited degenerative changes, including loss of the plasma membrane, cytoplasmic organelle (mitochondria, granular cytoplasmic network) disintegration, and the release of granules among collagen fibrils (Figure 4C).

The released granules exhibited a spectrum of ultrastructural states, reflecting varying degrees of activation and release. These included (1) intact, electron-dense granules with a preserved membrane; (2) granules with partial membrane loss and reduced electron density, suggesting partial secretome release; and (3) swollen granules with an electron-lucent, finely dispersed content enclosed by their membrane (Figure 4C).

The granules of degraded MCs’ fragments were directly associated with collagen fibrils, demonstrating discrete niches in the extracellular matrix enriched with MC secretome components (Figure 5C).

Additionally, MCs established direct contacts with both peritubular myoid cells and TCs—a stromal fibroblast-like cell type recently characterized in the peritubular and intertubular space of the testicles. TCs were identified by their distinctive long cytoplasmic extensions, or telopodes. These processes comprised alternating podomers—dilated portions frequently harboring mitochondria—and podoms, which are the extremely thin intervening segments (Figure 5D).

**Figure 6:**
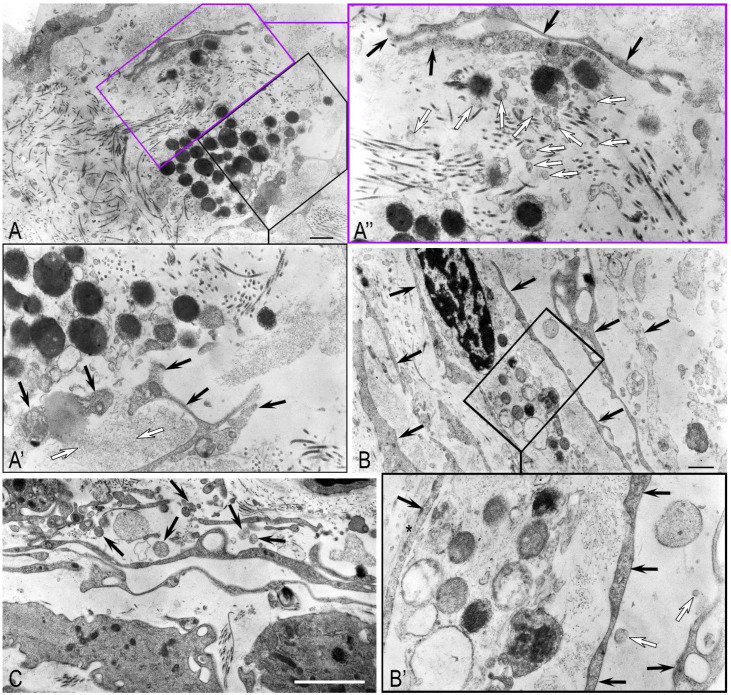
Ultrastructural organization of the testicular interstitium from a patient with AZFc deletion: (**A**) A degranulating mast cell (MC) surrounded by telocyte telopodes. (**A’**) A higher-magnification view of the area in (**A**), showing telopode fragments (black arrows) contacting MC granules and numerous microvesicles (white arrows) in the extracellular space. (**A”**) Detailed view of another region from panel (**A**), illustrating telopode fragments (black arrows) adjacent to MC granules. Note the compartmentalization of the local tissue niche, with foci of collagen fibrillogenesis (white arrows). MC secretory granules exhibit varying degrees of degranulation, indicated by regions of reduced electron density. (**B**) The interface between telopodes and an MC (black arrows). (**B’**) A higher-magnification view of the area outlined in panel (**B**), showing a contact site (asterisk) between a telopode (black arrow) and the MC. Microvesicles (white arrow) are visible in the extracellular matrix. (**C**) Abundant microvesicles (black arrows) localized near telopodes. Scale bar: 1 µm.

The telopodes of TCs formed an extensive communication network, revealing direct homocellular contacts with each other and heterocellular contacts with both myoid cells and MCs. MCs were frequently surrounded by these telopodes and often observed in a state of degranulation. Most significantly, the extracellular space surrounding these interactions was rich in microvesicles, which were abundantly released by MCs and located among MC granules near telopodes (Figure 6A–C). This ultrastructural evidence suggests the formation of a complex stromal reticular network that integrates multiple cell types via physical contacts and vesicle-based signaling.

Leydig cells, the endocrine component of the testicular interstitium, are pivotal for male reproductive function. Residing individually or in small clusters between seminiferous tubules, they synthesize key androgens—including testosterone, dihydrotestosterone, and estradiol—and their function can be influenced by various trophic hormones.

**Figure 7:**
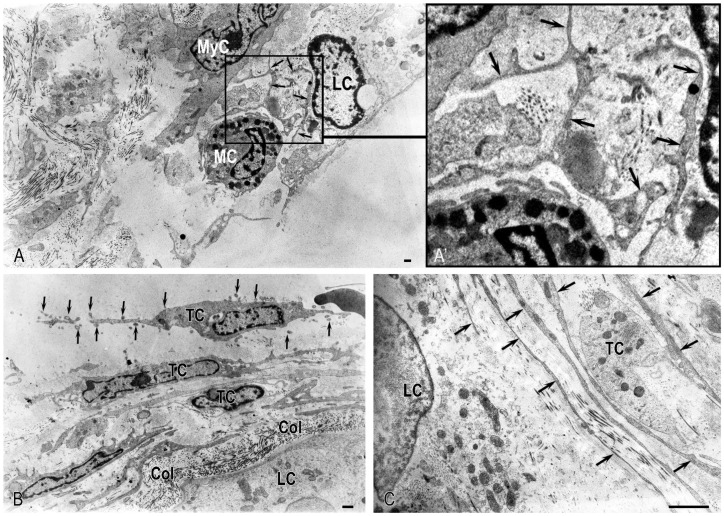
The stromal component of the intertubular interstitium in a patient with AZFc deletion: (**A**) Electron micrograph showing spatial relationships between a Leydig cell (LC), a myoid cell (MyC), an MC, and telocyte processes (arrows). (**A’**) A higher-magnification view of the area outlined in panel (**A**). (**B**) A Leydig cell surrounded by collagen fibrils (Col) and elongated telocyte (TC) processes. Arrows indicate numerous exosomes in the vicinity. (**C**) A degenerating Leydig cell (LC) encompassed by telocyte (TC) processes (arrows). Method: Transmission electron microscopy. Scale bar: 1 µm.

In the interstitium, Leydig cells were sparsely distributed as single cells or, rarely, in small clusters of 5–7 cells (Figure 2D–F,M and Figure 3B). Ultrastructural examination revealed that the Leydig cells’ cytoplasm was electron-lucent and organelle-sparse. The smooth endoplasmic reticulum was vestigial, consisting of small, non-anastomosing vesicles (Figure 7A–C). Mitochondria were small, polymorphic, and electron-dense, frequently exhibiting disorganized cristae (Figure 7B,C). These features—particularly the underdeveloped endoplasmic reticulum and abnormal mitochondrial architecture—represent key morphological correlates of steroidogenic function.

The ultrastructural profile of Leydig cells indicated low steroidogenic activity. Mitochondria with tubulo-vesicular cristae, a hallmark of steroid-producing cells, were seldom encountered in our samples. Notably, the few such mitochondria detected were predominantly within degenerating cells, as evidenced by the loss of the plasma membrane and the extrusion of cellular contents into the intercellular space (Figure 7C).

No well-defined Golgi complex or rough endoplasmic reticulum was observed in the cells. The nuclei were large, rounded, and often eccentrically displaced, with light nucleoplasm suggesting intracellular edema. The chromatin was predominantly marginated along the nuclear envelope, with additional clumping in the nucleoplasm. A subset of cells contained one or two prominent nucleoli, with the latter being less common. A local expansion of the perinuclear space was evident (Figure 7A).

Collectively, these findings demonstrate that Leydig cells displayed dystrophic alterations of varying degrees, including overt destructive changes.

## 3. Discussion

These clinical observations demonstrate that secretory azoospermia associated with AZFc deletion coincides with a diminished testicular reserve. Infertility progression is likely driven by fibrous thickening of the seminiferous tubule walls, which compromises the blood–testis barrier [62,63]. Regardless of etiology, peritubular fibrosis represents a consistent and reliable histological indicator of male infertility [36,44].

Testicular MC infiltration is an established feature of the testicular microenvironment in infertile men, particularly in idiopathic azoospermia [36,41,46]. Peritubular sclerosis may be driven by MC activation, as previously suggested. According to this model, MC-derived tryptase enhances chemotaxis and fibroblast activation, thereby stimulating collagen synthesis [36,64]. Furthermore, tryptase—similarly to histamine—induces fibroblast-to-myofibroblast differentiation, thereby accelerating fibrotic progression [65,66,67].

Chymase contributes directly to fibrogenesis by cleaving type I procollagen to promote collagen fibril assembly [68,69]. This aligns with the findings of Hussein MR et al. (2005), who demonstrated that elevated MC levels drive collagen deposition in testes with impaired spermatogenesis [70]. Taken together, the evidence suggests that specific proteases released by activated MCs are implicated in the etiology of peritubular fibrosis, including carboxypeptidase A3 [71,72].

Microdeletions of the Y chromosome are a well-established genetic cause of impaired spermatogenesis [15,73,74,75], typically presenting as azoospermia or severe oligozoospermia. The long arm of the Y chromosome harbors three critical azoospermia factor (AZF) subregions—AZFa, AZFb, and AZFc—within intervals 5 and 6. Deletions may encompass the entire AZF locus or individual subregions, usually resulting in infertility. The prevalence of these microdeletions varies geographically and ethnically, affecting approximately 13–18% of men with idiopathic infertility, 5–10% of those with azoospermia, and 2–5% of those with oligozoospermia.

The prevalence and histological manifestations of AZF microdeletions vary by subregion. AZFa deletions (∼5% of cases) typically present with a Sertoli-cell-only (SCO) phenotype [76] or, less commonly, focal spermatogenesis. Complete AZFb deletions (10–16% of cases) result in azoospermia, histologically characterized by SCO or spermatogenic arrest [77]. In contrast, AZFc deletions are the most frequent (∼80% of cases) [78]. While often leading to azoospermia, sperm can be successfully retrieved via TESE in approximately 50% of these patients [12]. The histopathological hallmarks of AZFc deletion include spermatogenic maturation arrest [77].

This study provides the first ultrastructural visualization of testicular TCs and their spatial association with MCs and Leydig cells in the context of AZFc deletion. Notably, the resident MCs were functionally active, according to their ultrastructural organization, and formed contacts with both TCs and Leydig cells. Conversely, the Leydig cells themselves exhibited sparse organelles, and a subset displayed degenerative changes, indicating compromised function. TCs, a recently identified stromal cell type with a unique ultrastructure, are present in the reproductive system [79,80,81,82,83,84,85]. Within the testicular stroma, we observed an extensive network formed by intercellular contacts between neighboring TCs and their long projections (telopodes).

Our findings indicate that TCs can play an important role in testicular stromal homeostasis by establishing both homo- and heterocellular contacts [86,87,88,89]. Specifically, TCs can coordinate MC activity, as evidenced by the frequent degranulation of MCs in direct contact with telopodes. This interaction may be facilitated by TC-derived vesicles, which we observed in the extracellular matrix among collagen fibrils. These microvesicles are likely carriers of bioactive molecules, representing a mechanism for paracrine signaling and tissue remodeling within the testicular microenvironment [90].

For the comparison of our results, we selected patients with NOA without genetic abnormalities as the control group [52]. Our preliminary data suggest that in the absence of such genetic anomalies, patients with NOA exhibit a greater number of mast cells in the peritubular stroma. Thus, testicular fibrosis in non-obstructive azoospermia caused by AZFc deletions appears to be one of the factors promoting male infertility. At the same time, in subsequent studies, a thorough qualitative and quantitative analysis of the intercellular cooperation intensity between MCs and TCs is needed to facilitate a more objective establishment of the role their interaction plays in the pathogenesis of male fertility disorders, including non-obstructive and obstructive azoospermia.

The main limitation of this study is the relatively small sample size (*n* = 14), which is due to the rarity of isolated AZFc deletion as a cause of non-obstructive azoospermia, as well as the strict inclusion criteria (verified deletion, absence of other genetic disorders, and a unified material collection protocol). The small sample size limits the ability to conduct extensive multifactorial statistical analysis and requires caution when extrapolating the findings to the entire population of patients with AZFc deletion. Nevertheless, the homogeneity of the study group in terms of the key etiological factor allowed us to minimize the influence of confounding factors and identify specific ultrastructural patterns that are characteristic of this genetic abnormality. The study did not include a separate control group of healthy fertile men (normozoospermia) due to the obvious ethical limitations of obtaining biopsy material from testicular tissue in this category of individuals. To partially overcome this limitation, we used the following approach: comparing the obtained data with reference values of hormonal indicators.

The priority of the work was a detailed qualitative description of the ultrastructural organization of intercellular interactions (in particular, in the “mast cell–telocyte” system) using transmission electron microscopy, the “gold standard” for identifying TCs. This determined the design of the study as predominantly descriptive.

Despite the listed limitations, the data obtained provide fundamentally new information on the ultrastructural organization of the seminal tubule microenvironment in AZFc deletion and demonstrate for the first time the potential role of TCs in mast cell activity regulation.

## 4. Materials and Methods

### 4.1. Case Selection

This study included 14 men with non-obstructive azoospermia (karyotype 46XY, AZFc deletions). The ages of the patients ranged from 24 to 52 years. Biopsies were obtained during TESE surgery at the Center for Reproductive and Cellular Medicine of the State Budgetary Institution Krasnodar City Clinical Hospital. The main criteria for selecting patients were the presence of azoospermia (absence of spermatozoa in the ejaculate), genetic disorders (AZFc deletions), and one or more failed attempts to participate in assisted reproductive technology protocols (IVF/ICSI). The exclusion criteria were acute inflammatory diseases of the reproductive tract, severe metabolic disorders, systemic diseases, and varicocele.

### 4.2. Tissue Probe Staining

The tissue probes left after the routine diagnostic procedure were fixed in buffered 4% formaldehyde and embedded in paraffin. Paraffin tissue sections (5 and 2 µm thick for histochemical and immunohistochemical staining, respectively) were deparaffinized with xylene and rehydrated with graded ethanol according to a standard procedure [91]. Tissue probes of approximately 1 mm^3^ were fixed in 2.5% glutaraldehyde and 1% osmium tetroxide solutions and analyzed using electron microscopy [91].

### 4.3. Immunohistochemistry and Histochemistry

For the immunohistochemical assay, we subjected deparaffinized sections to antigen retrieval by heating the sections in a steamer with R-UNIVERSAL Epitope Recovery Buffer (Aptum Biologics Ltd., Southampton, UK) at 95 °C × 30 min [92]. After antigen retrieval and, when required, endogenous peroxidase quenching, the sections were incubated with primary antibodies. IHC studies were performed with antibodies specific to mast cell proteases, including tryptase (Anti-Mast Cell Tryptase antibody [AA1]), mouse monoclonal antibody, #ab2378), chymase (Anti-Mast Cell Chymase antibody [CC1], Mouse Recombinant Monoclonal antibody, #ab2377), carboxypeptidase A3 (Rabbit polyclonal, #ab251696, Cambridge, UK), and αSMA (Mouse monoclonal [1A4], #ab7817, Abcam, Cambridge, UK). Secondary goat anti-mouse or anti-rabbit antibodies (AmpliStain anti-Mouse 1-Step HRP or AmpliStain anti-Rabbit 1-Step HRP [SDT GmbH, Baesweiler, Germany]) were employed for monoplex immunohistochemical detection of molecular targets using the DAB Peroxidase Substrate Kit (Vector Laboratories, Burlingame, CA, USA) according to the manufacturer’s instructions. For fluorescence detection, the following secondary antibodies were used: Goat anti-rabbit IgG Ab (Alexa Fluor^®^ 488), #ab150077, Abcam, UK; Goat Anti-Mouse IgG H&L (Alexa Fluor^®^ 488), #ab150113 Abcam, UK; and Goat Anti-Mouse IgG H&L (Alexa Fluor^®^ 555), #Ab150114 Abcam.

Histochemical staining with Mayer’s hematoxylin and eosin was performed according to the manufacturer’s instructions.

### 4.4. Controls

Control incubations were performed by omitting primary antibodies or substituting primary antibodies with the same IgG species (Dianova) at the same final concentration as the primary antibodies. The exclusion of either the primary or secondary antibody from the immunohistochemical reaction and the substitution of primary antibodies with the corresponding IgG at the same final concentration resulted in a lack of immunostaining. Specific and selective staining of different cells using primary antibodies from the same species on the same preparation is a sufficient control for immunostaining specificity.

### 4.5. Electron Microscopy

To conduct an EM study, pieces of about 1 mm3 in size were cut from the surgical materials and then fixed in a 2.5% glutaraldehyde solution and 1% osmium (VIII) oxide solution. Then, the material was dehydrated in alcohols of increasing concentrations, soaked in a mixture of “propylene oxide–araldite resin”, covered with araldite resin, and then placed in a thermostat at 60 °C for 48 h. After analyzing the light-optical samples (section thickness 1.0–1.5 microns, dyed with toluidine blue), the sites for ultramicrotomy were carefully selected.

Ultrathin sections with a thickness of 100–120 nm were cut out on an LKB ultramicrotome (Bromma, Sweden). Sections were stained with uranyl acetate and lead citrate [91]. The ultrastructural study of samples was performed using JEM-2100 and JEM 100-CX electron microscopes (JEOL, Tokyo, Japan) in transmission mode at an accelerating voltage of 80 KV.

Transmission electron microscopy is the “gold standard” for TC identification [93,94,95]. The ultrastructures of mast cells and TCs have fundamental differences. If the ultrastructure of mast cells has been well known for a long time, then the ultrastructural features of TCs can be characterized as follows:

TCs have a characteristic ultrastructure that distinguishes them from other cells, including mast cells: a TC is a process cell. Long branching processes (telopods) are one of the most characteristic morphological features of a TC. The number of processes extending from the cell body, determined on a single slice, can vary from one to five, more often two or three, and their length ranges from tens to hundreds of microns. Their thickness is uneven, with local extensions, but at the point of departure from the cell body, the thickness of the process is the smallest (0.5 microns). The dichotomous branching of the appendages forms labyrinthine networks. TC processes are called moniliform (“warty”, with extensions and constrictions, like beads on a string) due to the characteristic alternation of expanded and narrow sections in publications. The expanded sections are called podomes, and the narrow ones are called podomers [96]. The width of the podomes is 250–300 nm, and the width of the podomers is about 80 nm. The cytoplasm of podomes contains mitochondria, endoplasmic reticulum, and microbubbles. The number of microbubbles in the podomes is significantly higher than in the cell body. Absence of secretory granules in the cytoplasm. Other cells do not have a similar ultrastructure.

### 4.6. Image Acquisition

Stained tissue sections were observed using a Zeiss Axio Imager.Z2 equipped with a Zeiss Alpha Plan-Apochromat objective 100×/1.46 Oil DIC M27, a Zeiss Objective Plan-Apochromat 150×/1.35 Glyc DIC Corr M27, and a ZEISS Axiocam 712 color digital microscope camera (Zeiss, Jena, Germany). Captured images were processed with the software programs “Zen 3.0 Light Microscopy Software Package,” “ZEN Module Bundle Intellesis & Analysis for Light Microscopy,” and “ZEN Module Z Stack Hardware” (Carl Zeiss Vision, Jena, Germany) and submitted with the final revision of the article at 300 DPI. Images from the JEM 100-CX microscope were captured on film, and the negatives were analyzed after digitization using an Epson Perfection V850 Pro scanner (Seiko Epson Corporation, Suwa-shi, Japan).

### 4.7. Statistical Analysis

All absolute numerical values are presented as the mean values and the standard deviation from the mean (M ± SD). Statistical data processing was performed using the Statistica 8.0 software package (StatSoft, Tulsa, OK, USA). The normality of the distribution of quantitative variables was checked using the Shapiro–Wilk test. To compare the indicators with reference values, we used the one-sample Student’s *t*-test (for data with a normal distribution) or the one-sample Wilcoxon test (for distributions other than normal). The differences were considered statistically significant at a *p*-value of <0.05.

## 5. Conclusions

Based on our findings, we conclude that TCs can play a crucial regulatory role in the testicular interstitium. Our data suggest two potential mechanisms by which TC dysregulation may contribute to impaired spermatogenesis.

First, TCs may modulate the MC population and activity via exosomal microRNA, indirectly influencing Leydig cell function. As Leydig cells are the primary source of testosterone, their impaired viability—potentially triggered by aberrant MC interactions—could directly disrupt spermatogenesis.

Second, interactions between MCs and TCs, as well as the association of MC secretory granules with sites of tropocollagen and collagen microfibril accumulation, may enhance collagen fibrillogenesis and stimulate the deposition of the fibrous extracellular matrix in the interstitial space of the seminiferous tubules.

In summary, the present work provides novel insights into the testicular pathology associated with AZFc deletion, revealing a complex cellular interplay within the seminiferous tubule interstitium. This study highlights the potential role of TCs as central communication hubs that employ multiple mechanisms to integrate the activities of MCs, Leydig cells, and fibroblasts, thereby orchestrating tissue homeostasis and influencing spermatogenic outcomes.

## Figures and Tables

**Table 1:** Characteristics of hormonal indicators.

Indicator	Average Value	Reference Values	*p*
FSH (follicle-stimulating hormone)	17.2 ± 1.9 mIU/mL	1.5–12.4 mIU/mL	<0.01
LH (luteinizing hormone)	8.1 ± 2.4 mIU/mL	1.7–8.6 mIU/mL	>0.05
Total testosterone	4.7 ± 1.2 ng/mL	3.0–10.0 ng/mL	>0.05
SHBG (sex hormone-binding globulin)	28.3 ± 7.2 nmol/L	13–71 nmol/L	>0.05
Prolactin	11.6 ng/mL	2.0–18.0 ng/mL	>0.05
Estradiol	24.8–32.9 pg/mL	11.3–43.2 pg/mL	>0.05
Inhibin B	76.9 ± 21.7 pg/mL	147.0–364.0 pg/mL	<0.01

## Data Availability

The original contributions presented in this study are included in the article. Further inquiries can be directed to the corresponding author.

## Abbreviations

The following abbreviations are used in this manuscript:

MCs	Mast cells
ICH	Immunohistochemical
EM	Electron microscopy
STS	Sequence-tagged sites
IL	Interleukin
TNFα	Tumor necrosis factor alpha
CPA3	Carboxypeptidase A3
α-SMA	α-Smooth muscle actin
TCs	Telocytes
